# Generating demand for and use of evaluation evidence in government health ministries: lessons from a pilot programme in Uganda and Zambia

**DOI:** 10.1186/s12961-017-0250-4

**Published:** 2017-10-02

**Authors:** Sophie Witter, Andrew Kardan, Molly Scott, Lucie Moore, Louise Shaxson

**Affiliations:** 1 International Health Financing and Health Systems, Queen Margaret University, Edinburgh and Oxford Policy Management Associate, Oxford, UK; 2 Oxford Policy Management, Oxford, UK; 3 ODI, London, UK

**Keywords:** Theory-based evaluation, Evidence-based policy, Capacity-building, Health programmes, Zambia, Uganda

## Abstract

**Background:**

The Demand-Driven Evaluations for Decisions (3DE) programme was piloted in Zambia and Uganda in 2012–2015. It aimed to answer evaluative questions raised by policymakers in Ministries of Health, rapidly and with limited resources. The aim of our evaluation was to assess whether the 3DE model was successful in supporting and increasing evidence-based policymaking, building capacity and changing behaviour of Ministry staff.

**Methods:**

Using mixed methods, we compared the ex-ante theory of change with what had happened in practice, why and with what results (intended and unintended), including a qualitative assessment of 3DE’s contribution. Data sources included a structured quality assessment of the five impact evaluations produced, 46 key informant interviews at national and international levels, structured extraction from 170 programme documents, a wider literature review of relevant topics, and a political economy analysis conducted in Zambia.

**Results:**

We found that 3DE had a very limited contribution to changing evidence-based policymaking, capacity and behaviour in both countries as a result of having a number of aspirations not all compatible with one another. Co-developing evaluation questions was more time-consuming than anticipated, Ministry evidence needs did not fit neatly into questions suitable for impact evaluations and constricted timeframes for undertaking trials did not necessarily produce the most effective results and value for money. The evaluation recommended a focusing of objectives and a more strategic approach to strengthening evaluative demand and capacity.

**Conclusions:**

Lessons emerge that are likely to apply in other low- and middle-income settings, such as the importance of supporting evaluative thinking and capacity within wider institutions, of understanding the political economy of evidence use and its uptake, and of allowing for some flexibility in terms of programme targets. Fixating on one type of evidence is unhelpful in the context of institutions like ministries of health, which require a wide range of evidence to plan and deliver programmes. In addition, having success tied to indicators, such as number of ‘policy decisions made’, provides potentially perverse incentives and neglects arguably more important aspects such as incremental programmatic adjustments and improved implementation.

**Electronic supplementary material:**

The online version of this article (doi:10.1186/s12961-017-0250-4) contains supplementary material, which is available to authorized users.

## Background

Evidence can shape policy decisions in a variety of ways. Our primary interest is often in understanding whether and under what circumstances robust evidence directly contributes to a policy decision. Yet, as Johnson describes [1], the application of evidence to inform concrete policy decisions (such as scaling up, discontinuing or redesigning a particular programme) is only one aspect of its possible influence. In addition to this ‘instrumental’ role, how evidence is used in decision-making may also be classified as legitimising (using evidence to justify a prior decision), conceptualising (providing new ideas), symbolising (emphasising strategic points such as value for money) or mis-using (suppressed or used to serve a political objective) [2]. These different ways of using evidence may occur at different points in the policy cycle [1, 3], depend on the particular types of evidence available, and be influenced by wider organisational and contextual considerations [4].

The literature suggests that the extent of uptake of evaluation evidence (meaning evidence generated by all types of evaluations, but excluding routine monitoring) in policy is often relatively modest. A comprehensive report on relevance, quality and influence of impact evaluations conducted by the World Bank Group found that, while impact evaluation evidence was observed in some cases to make a positive contribution to development practice and policy debate, systematic use of evidence was weakened by a number of constraints, such as lack of government demand or champions [5]. This conclusion was echoed by a European Commission study on the effects of knowledge generated by EuropeAid’s strategic evaluations, which found that, although there were some notable cases of the information being used to inform distinctive policy choices or raise conceptual understanding, findings do not tend to be incorporated into decision-making at an institutional level without a concerted effort to ‘broker’ the evidence into the decision-making process [2].

Both reviews note that detecting or measuring the extent of evidence uptake is challenging, especially given that some of the broader level influences on how knowledge is used in the policy process may be intangible such as relationships between researchers, departments and wider stakeholders [4, 6, 7]. However, much can still be said about the factors that may affect the likelihood that relevant evidence is used in policymaking in a rational way. Focusing on the use of evaluation evidence in particular, and drawing on Johnson et al. [1], we categorise these factors as characteristics of the evaluation, characteristics of the evaluation user and wider contextual factors.

### Evaluation (supply) characteristics

In order to be relevant, information presented to policymakers should be salient (relevant to the problem), credible (high technical quality) and legitimate (produced without bias or political interference and through a transparent process) [8, 9]. One of the primary findings to emerge in the literature is that evidence produced by evaluations is not always perceived as relevant to the practical requirements of policymaking [2, 10–12]. Evaluations should address identified policy needs, deliver clear recommendations and pay close attention to political and contextual factors such as organisational culture [13]. Yet, evaluation evidence can appear to cater more to a research audience than the practical needs of policymakers. Impact evaluations in particular may be more focused on what happened in interventions rather than why the results arose, deliver recommendations that have many caveats (and are therefore not straightforward to interpret or apply), or present results in a highly technical way [2, 11]. Consideration of the political climate or the likely resource requirements of implementing recommendations may also be overlooked, according to the review of five case studies undertaken on behalf of the Centres for Learning on Evaluation and Results initiative (CLEAR) in 2014. The outcome is that policymakers often struggle to draw lessons from existing evaluation work or to locate evidence that meets their information needs.

Some policy questions that governments are concerned with are also not well suited to ostensibly rigorous evaluation techniques such as randomised controlled trials (RCTs) [11, 14], either because the methodology only answers a small part of the overall question or because data quality is poor. This means that what researchers define as rigorous evidence is not what is required to make policy decisions, raising issues of what constitutes ‘credible’ evidence.

Secondly, evaluation evidence is often not available when policy decisions need to be made [2, 10, 11]. The rapid decision-making that may be required by political calendars is incompatible with in-depth evaluation processes where recommendations may take several months or years to produce [14]. This means that, in common with other forms of evidence, windows of opportunity to influence policy may often be missed, and by the time results are available, evaluations may have lost much of their relevance to current policy issues.

A third factor identified in the literature is that empirical evidence is not well communicated to policymakers since reports may be excessively technical [12, 15] or they may be poorly written and ineffectively disseminated to their intended audiences [2].

The failure of some evaluators to deliver high quality, timely, policy-relevant and appropriately communicated findings to policymakers points to a wider concern that the priorities of the producers and users of evaluation are not closely aligned. This is in part due to the fact that evaluations in lower- and middle-income countries tend to be commissioned by international development partners [16]. There is some evidence that this is changing in line with the recommendations of the Paris Declarations, which call for greater in-country ownership of monitoring and evaluation (M&E) efforts. Yet, although there are some notable and important examples of evaluations being managed internally by government ministries with designated M&E oversight [13, 17], the study on supply and demand for evaluation evidence undertaken on behalf of CLEAR finds that in-country M&E work remains mostly limited to performance monitoring rather than evaluation [16]. As a result, evaluation evidence may often be generated by independent researchers who do not have policy concerns at the forefront of their agenda, and instead prioritise research objectives such as obtaining publication in peer-reviewed journals [10].

### User (demand) characteristics

A prerequisite for the actual use of evaluations in policy is that key political actors demand evidence to be made available and are receptive to the findings. Guidance produced by the World Bank Group on strengthening government capacity in generating and using M&E evidence argues that weak demand for evaluation evidence can pose an even greater limitation to the use of evaluation evidence in policy than the issues around evaluation characteristics described above [17, 18].

There are mixed findings in the literature on whether this demand exists in different contexts. Hyder et al. [19] find that policymakers actively value the evidence produced by evaluations. CLEAR also note, in their study of M&E systems in nine sub-Saharan African countries, that there are promising and increasing indications of evaluation demand by governments and civil society [16, 17]. However, low demand has been a commonly encountered obstacle across the body of the World Bank Impact Evaluation Group’s work [18].

Weak demand for evidence is partly to do with issues related to evaluation characteristics, but there are also important causes unrelated to the supply of impact evaluations. In the first place, evaluation evidence may simply be less useful to policymakers under some circumstances than other kinds of knowledge. Second, the type of evaluation matters; in South Africa, the National Evaluation System conducts implementation, impact, diagnostic, design and economic evaluations to help “*maximise the likelihood of alignment between the evaluation and departmental willingness to use the findings*” ([17], p. 5). Additional evidence sources that policymakers may demand include the accumulated experience of stakeholders and institutions, wider research evidence, statistical data, and the knowledge of citizens and stakeholders about their own policy needs [12, 20]. A balance of different sources and types of evidence is required to develop good policy, and it is not the case that evaluation evidence is self-evidently superior.

The values and beliefs of individual decision-makers can also be strong drivers of uptake [3, 20]. Their assumptions about what constitutes robust evidence can be difficult to overturn, particularly where new information contradicts a strongly held ideology [5]. The propensity of policymakers to rationally apply evidence to policy issues also depends crucially on political calculations and contextual factors. Evidence may be disregarded or even concealed if it is not consistent with a particular political calculation or threatens the interests of powerful groups [20].

Another potential cause of limited demand for evaluation evidence is that policymakers themselves are not sufficiently skilled in evaluation methods, in part due to limited exposure [5]. The CLEAR midterm evaluation report [16] observed that demand for evaluations by policymakers is often ‘latent’ – they do want information to support their decisions but do not recognise that evaluations can be a source of this evidence. In a systematic review of the barriers to evidence use in policymaking, Oliver et al. [10] report that policymakers themselves expressed a need for support in building their own knowledge to help them make use of evaluation evidence.

### Contextual factors relevant to evidence uptake

Beyond the immediate characteristics of evaluations and evaluators, the literature also emphasises the role of political, institutional and organisational factors in shaping the way that evidence is used in policymaking. A central argument is that, if evaluations explicitly engage with the political economy, the evidence is more likely to be used [16]. However, there are some acknowledged challenges associated with assessing political economy factors. Political systems are complex and difficult to characterise, and Liverani et al. [21] note that there are several gaps in the current understanding of the implications of different political systems for evidence uptake. However, there have been some recent advances in developing knowledge in this area [1, 16].

Among the findings is that the distribution of decision-making power across the political system has a crucial effect on the opportunities for evidence uptake. In more mature democracies, decentralised systems, in which many actors have a stake in guiding policy, may be associated with greater use of evidence to support processes of policy contestation, i.e. the ability to marshal evidence becomes important as a way to secure support for particular policy positions or undermine competing views [20, 21]. In a related point, some studies argue that higher levels of government accountability observed in mature democracies can lead to increased evidence use since policymakers face pressure to demonstrate and justify the basis on which decisions are taken [17, 22].

Although the existence of political accountability and platforms for policy debate may create potential for evidence uptake, they do not guarantee it. Instead of applying evidence to policy problems in an impartial way, political actors may behave opportunistically by purposefully selecting evidence to back up pre-existing policy positions or present findings in a misleading way [20, 21]. Where accountability to electorates and civil society is strong, the motivation to rely on the evidence basis for policy choices may be outweighed by the pressure to meet public perceptions or fulfil election promises [11]. There is no system of government that ensures ‘rational’ evidence-based policy [23] because the nature of politicisation varies between contexts, systems and issues.

Several papers also identify features of individual government ministries that are relevant to evidence use. Liverani et al. [21] report that highly fragmented responsibility within individual bureaucracies can reduce the ability of ministers and their staff to engage with evidence that falls outside their immediate area of work [21]. A high rate of staff turnover is also found to lower the potential for critical engagement with new evidence by shortening the ‘institutional memory’ of the department, failing to use existing evidence and causing current practices to appear novel. Organisational structures and processes are also important; Shaxson et al. [4] observe how departmental planning, budgeting and reporting processes may focus the evidence base on the short term rather than more strategic issues and consequently influence the relationships between the various groups of people who provide, broker and use evidence.

### The pilot programme

The Demand-Driven Impact Evaluations for Decisions (3DE) pilot was designed by the Clinton Health Access Initiative (CHAI) and IDinsight. It was based on the recognition that Ministry of Health (MoH) officials often lack evidence on the most effective and efficient ways in which to deliver known clinical interventions and services. Further, where evidence is generated, it is often not relevant to the operational needs of MoH officials or done within a period that meets decision-making timeframes. The 3DE pilot model aimed to facilitate a more demand-driven approach to the evaluation of health interventions by (1) identifying relevant, suitable and priority evaluation questions from the ministries, (2) conducting these evaluations rigorously but rapidly and in an affordable manner, and (3) catalysing the response to their findings and sharing the lessons learned from this process more widely, so as to influence future evaluation processes. Under this pilot, 3DE was expected to conduct eight (later revised to five) impact evaluations that influenced managerial decisions in six instances (later revised to four) in Uganda and Zambia. The interventions that were evaluated under the 3DE pilot are summarised in Table 1. The evaluations were expected to be completed and presented to policymakers within 9 months of their commencement. The pilot had a budget of £2 million and was funded by the UK Department for International Development.

**Table 1:** Interventions evaluated under the 3DE pilot

Intervention name	Country	Description
Mama kits	Zambia	This intervention provided non-monetary ‘mama kits’ to pregnant mothers conditional on delivering at a health facility. The objective of the intervention was to increase facility delivery rates, and ultimately maternal and newborn health outcomes in Zambia
Community-fixed point implementation of insecticide-treated bed-nets (ITNs)	Zambia	The intervention distributed ITNs to community members using a ‘fixed point’ approach, in which bed-nets were given out at a particular place in the community rather than by volunteers visiting households door-to-door to deliver and hang-up nets; the objective of the evaluation was to understand how this approach compared to the door-to-door method in terms of rates of retention and use of bed-nets, and cost-effectiveness
Health facility reinforcement and early infant diagnosis (EID) and immunisation service integration on HIV testing and immunisation services	Zambia	The objectives of the programme were to improve the identification of HIV-positive mothers and infants in Zambia, in order to ultimately improve the timeliness of treatment and health outcomes It strengthened supplies of HIV testing kits, reinforced guidelines around early-infant diagnosis and HIV-testing for mothers to health service providers, and also included a component that sought to integrate EID testing services with scheduled 6-week immunisation visits for infants
Decongestion of busy anti-retroviral therapy (ART) clinics	Zambia	The objective of the intervention was to improve ART service efficiency, and ultimately the supply of ART; it involved providing resources to improve the availability of stocks at health facilities, emphasising the application of existing guidelines around ART refills, and providing a designated person to work in targeted facilities to ensure that protocols are correctly observed
Family clinic days	Uganda	The intervention provided family-centred care and health education to HIV-positive adolescents and children and their families, through a designated clinic day; during these, clinics delivered specialised health education and psychosocial support to patients and caregivers; the main objectives were to improve the retention of HIV-positive paediatric and adolescent patients in care

This article presents findings from the evaluation of the 3DE pilot. Its main objective is to provide lessons on how to design evidence support programmes or initiatives for the health sector (and potentially other sectors too). It builds on the small but growing literature documenting attempts to support evidence-based policy at institutional level in low- and middle-income countries.

## Methods

The overall aim of the evaluation, conducted in 2015, was to assess whether the 3DE model had been successful in supporting and increasing evidence-based policymaking, building capacity and changing the behaviour of Ministry staff in terms of demanding and using evidence (as these were the 3DE objectives).

### Analytical approach

**Fig. 1:**
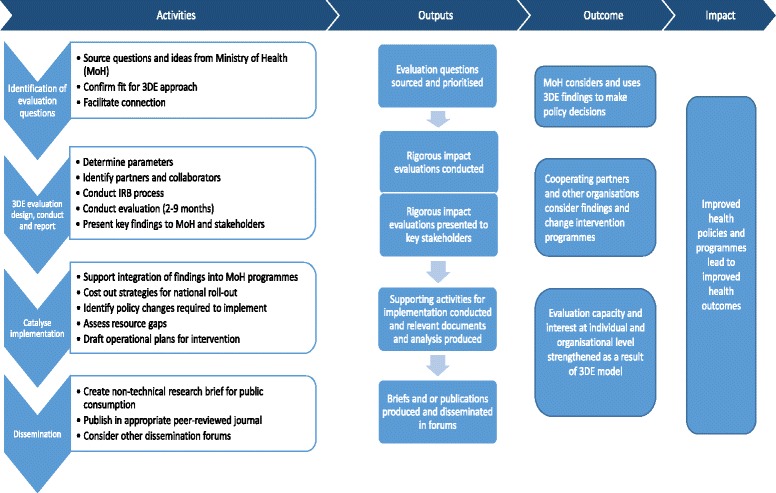
Programme theory of change

As the evaluation was commissioned ex-post and there was no credible counterfactual, the evaluation took a theory-based approach, starting from the theory of change for 3DE, structured by process, outputs and outcomes (Fig. 1). Assumptions and causal pathways were elaborated, based on the literature and initial discussions. These included behavioural outcomes, which were expected, hoped for or ideal, particularly relating to the outcomes of the programme (see extended theory of change). Mixed methods were then used to undertake contribution analysis and to establish, for each of the main domains, (1) what happened in practice (what activities were undertaken by 3DE and what the responses were of Ministry and other stakeholders); (2) why what happened took place (particularly the role of the 3DE intervention but also any other relevant factors); and (3) with what results (intended and unintended). These findings were then compared with what was planned in the original 3DE programme documents and the theory of change. The analysis answered evaluative questions about 3DE but also refined the theory of change for future programmes.

The evaluation considered 3DE as a whole, including consideration of how it was established and the prioritisation of evaluation questions that the 3DE evaluations would seek to answer. However, each individual evaluation conducted by 3DE constituted a case study within the overall framework, which could be compared in order to generate a richer understanding of differences and similarities.

As most of the work had been undertaken in Zambia, this was the focus of the evaluation; nevertheless, a modified set of questions was used in Uganda to learn from the experience there. The evaluation was therefore able to draw from evidence from two national settings and across five different evaluations.

### Data collection and analysis

Five main sources of evidence were used for the evaluation.

#### Quality assessment of five evaluations

Given the overall objective of the intervention, the purpose of this component was to test the effectiveness of the programme in generating high quality evaluations. The quality of the 3DE evaluations was assessed against a set of specific questions, including the relevance and clarity of the evaluation questions; the extent to which contextual factors that could affect the evaluation were considered; the clarity and level of detail with which the intervention was described; the extent the evaluation considered other programmes that may have affected key indicators; the appropriateness of the evaluation methodology, including the adequacy of the sample size, and the extent to which baseline differences, spillover effects, externalities, imperfect compliance, non-response and attrition were dealt with appropriately; the appropriateness and rigour of the data sources and data collection; the rigour of the analysis including appropriate calculation of standard errors and use of sampling weights; the clarity and plausibility of the links between data, interpretation and conclusions; and the external validly of the evaluation.

The assessment was applied to all five evaluations conducted by 3DE, although only three were finalised at the time of evaluation and so could be examined in relation to all quality assurance questions. Details of the assessment have been previously published [24].

#### Key informant interviews

Forty-six key informant interviews were conducted, using semi-structured topic guides. The participants included a range of stakeholders internationally and in the two focal countries from government (n = 17), the 3DE programme (n = 16), and development partners and other institutions (n = 13). The interviewees were initially sampled purposefully to maximise information-rich cases augmented by snow-balling during the evaluation. The interviews were thematically coded and entered into an analysis spreadsheet, which was structured according to the key nodes in the theory of change. By reading down the columns, views from informants and evidence from 3DE programme documents could be triangulated on each topic, and a summary of evidence created.

#### Document analysis

Over 170 documents of various types (such as 3DE programme documents, financial reports, MoH policies and strategies, and development partner reports) were read, thematically coded and entered into the same spread sheet used for the key informants [25]. An important caveat was that much of this evidence was generated by the project and may have therefore been biased toward demonstrating progress and success. Internal evidence was given weight in the summary description of what was done and why. External evidence was given more weighting in the final evaluative judgements.

#### Literature review

A literature review was undertaken to understand the background and wider global context for 3DE. This focused on a number of topics, including evidence on general experience and the efficacy of demand-led evaluations; evidence on evaluation use and uptake by policymakers; evidence on formal and informal barriers and enablers for conducting and using impact evaluations by policymakers/government officials; other global initiatives and experiences of demand-led evaluations; and reviews of on-going and recent impact evaluations in the health sectors of Zambia and Uganda.

The body of literature reviewed included research papers, articles, theoretical discussion papers and synthesis reports drawing together the findings of other work.

#### Political economy analysis

A political economy analysis of the health sector in Zambia was conducted to better understand the contextual factors influencing the outcomes of the 3DE model, with particular focus on resource allocation and decision-making within the two Ministries being studied, and how evidence was used in their policymaking processes.

### Evaluation limitations

Some important limitations should be noted, including the relatively short timeframe of 3DE, which did not allow for the assessment of health outcome changes or the necessary maturation of catalysation activities. As a result, the evaluation focused on processes more than outcomes. The Ugandan experience also received more limited analysis compared to the Zambian one, given that no evaluation had yet been completed in Uganda.

## Results

The theory of change of 3DE (Fig. 1) stipulated that findings from 3DE evaluations, that were based on questions raised by the MoH, were conducted in close collaboration with them, and that produced timely and well-presented results, were more likely to be adopted into policy than traditional evaluations which lacked these characteristics. Improved evidence uptake was expected to result in the implementation of better health policies and programmes that would ultimately improve health outcomes for the population. Supporting the MoH in following through with evaluations and assisting them in implementing its recommendations was seen, in addition to the relevance and timeliness of the evaluations, as an important feature of this pilot. All of these steps were examined in the evaluation of 3DE, alongside explanatory factors.

### Question sourcing

Although the approach of starting from MoH questions was laudable, one challenge faced was that ministries do not generally have a prioritised set of research questions and are often institutionally divided. In Zambia, a set of ministerial research priorities had been established but it was more of a theoretical wish list and many questions were not amendable to answer by impact evaluation. In Uganda, individual disease programmes had research agendas but these had not been prioritised across the ministry as a whole. Developing options and assessing them was a much more intensive process than anticipated by the 3DE programme, and depended on strong links to specific groups within the MoH, which also biased the selection towards certain favoured programmes (and away from areas with less strong programmes such as mental health). In Uganda, a number of ‘false starts’ were made with different units within the MoH, which is one reason why ultimately just one impact evaluation was delivered there.

Another challenge faced by 3DE implementers was identifying questions which met the rigorous criteria of the programme. These included that the intervention was feasible to implement in the timescale (9 months), had potential for wide impact, could be addressed through a rigorous experimental or quasi-experimental counterfactual-based impact evaluation, was being rolled out in a way and timeframe that could be used for the study, and where the chances of catalysing change in the future were strong. It is clear that these criteria were, in combination, very demanding, and this in part explained the difficulty of identifying successful questions in Uganda and reaching the targeted four evaluations in Zambia.

3DE implementers worked closely with Ministry partners (the MoH in both countries, but also the Ministry of Community Development, Mother and Child Health (MCDMCH) in Zambia) to source questions, but partners found it harder to engage in the prioritisation of questions that involved more technical issues about research design. This reduced their ultimate ownership in the process.

### Evaluation design, conduct and reporting

The research questions posed by the evaluations were all shown to address relevant healthcare challenges in Zambia and Uganda and, in at least one case, the evaluation was timed to meet an important opportunity (a large-scale bed-net distribution). The others addressed less urgent issues and took longer to conduct than originally prescribed. The rationale for the particular interventions evaluated in each study, including a description of underlying challenges and how the intervention mechanism was expected to address them, was not always well described in the evaluation reports.

The overall quality of the design of the 3DE evaluations was assessed as variable, with some weaknesses stemming from the constraints placed on the evaluations in terms of timeframes and budgets.

One aspect of the evaluation design that was consistently conducted well was the choice of primary outcome given the available study period. The evaluations all focused on measures that could be plausibly expected to change over a period of months if the intervention was effective. Although all evaluations did make an appropriate choice of primary outcome, there were some issues with the indicators used to track these outcomes. The evaluations were also well designed to make efficient use of the available budget and were organised to minimise additional workload for health staff by being aligned to a large degree with current practices in health facilities.

The overriding concern with the design of 3DE evaluations was that the findings were not easily generalisable to other contexts (i.e. there was a problem with the findings’ low external validity). Many of the evaluations were only able to cover a limited geographic area and a small sample due to the relatively low budget and timeframe to implement the evaluation. There was also a concern that some of the 3DE evaluations may not have delivered sufficient internal validity despite their randomised design, given the small size of the treatment groups. The time and budget constraints also affected the implementation of the interventions themselves, which in some cases may have been too ambitious for the short evaluation period.

In view of some of these concerns it was not clear that the choice of a given RCT always made the best use of the available budget. In some cases, a simple operational pilot or process study might have provided sufficient evidence around the implementation of interventions to help guide future programming decisions. This was particularly the case for interventions that sought to reinforce existing practices rather than providing new and previously untested solutions (such as the Early Infant Diagnosis simple intervention and the decongestion intervention).

The evaluations appeared to have collected good data using appropriate techniques. Where the data-collection processes were reported on, these processes were assessed as good. Sample sizes were an issue for some of the evaluations. Quantitative findings were for the most part presented well. The explanation and interpretation of results could, however, have been further developed, with the overall findings better situated within a broader discussion of the context and likely mechanisms involved.

### Dissemination and activities to catalyse implementation

We concluded that 3DE generally had a good awareness of entry points as well as of key stakeholders and disseminated evaluation findings well to key stakeholders, mostly to the implementers of the interventions they were evaluating. However, in order to provide rapid feedback, presentations preceded the finalisation of reports, which was problematic when final findings later changed. The ensuing ‘policy decisions’ (for the three completed evaluations) took the form of advisory notes. The implications of the evaluations were largely managerial, rather than implying larger changes in programming or resource allocation. There was also limited scope for ‘catalysation’ work (3DE providing supporting models, costing and plans for scale-up) and uptake had been limited at the time of evaluation. There was no resourcing for 3DE to follow-up on what had happened after ‘policy decisions’ were taken.

3DE did not have a specific capacity-building plan beyond working closely through the stages of the programme with MoH/MCDMCH partners. Interviews indicated that individuals who worked closely with 3DE did benefit in terms of capacity development. More broadly, there was an expression of latent demand for evidence, although not necessarily for evaluations specifically. Both ministries (MoH and MCDMCH) lacked a wider strategic approach to evidence and research, and there was no indication that this had changed as a result of 3DE.

Key stakeholders did not always have a clear understanding of the findings and raised questions about the external validity of results for other areas of the country and in ‘normal’ health system conditions. Ownership of findings was partial. Limited staff time, a lack of capacity in terms of research staff in key partner agencies, and a lack of incentives for evidence use were some of the factors behind this.

Although the timeframe of our evaluation did not permit assessment of outcomes, the expected impact was unlikely to be transformational. Some 3DE evaluations supported a reinforced implementation of the status quo. Others suggested potential for some cost savings, though only in some contexts in Zambia, and with careful attention to ensure replicability of results. Others suggested a potential saving but largely for donors.

### Explanatory factors

There were a number of issues relating to context and internal factors that contributed to the failure of the 3DE pilot to achieve its key objectives. Among the contextual factors, the lack of an effective strategic prioritisation of evidence-based decision-making within government was highlighted as a constraint, along with unclear ministry roles in Zambia (linked to the split of the MoH into two ministries in 2012).

The evidence that was deemed as most in need by Ministry officials in Zambia was operations research and situation analyses to identify and rectify bottlenecks in delivery or service and research to better understand the behaviour and motivation of end-users in regard to non-utilisation, as well as synthesis of evidence from other contexts. National reports also highlight insufficient use of data at all levels of government and further note that capacity and funding for research is limited [24]. Demand for impact evaluation evidence is generally low, although the health sector is seen by some as more advanced in this respect, with staff having been exposed to clinical trials during training. Government/donor relationships are also key to evidence uptake in contexts, like Zambia, where donor agreement and funding are crucial to the implementation of policy. This was well understood by 3DE, but managing these complex dynamics at various stages of the programme still required intensive efforts.

Internal programme factors included positive ones, such as a strong starting base for CHAI, which was well embedded in the MoH, as well as negative, such as an initial understaffing of the 3DE Uganda programme. The degree of involvement of the MoH Principal Investigators and co-Investigators also varied across the studies and contexts, as might be expected, according to individual time and interest; these affected ownership and legacy.

## Discussion

This evaluation adds to a small but growing body of evidence on how to support evaluation evidence uptake in the sub-Saharan African context. Mijumbi et al. [26], for example, examine factors affecting evidence uptake in Uganda, focussing on the degree of control which policymakers have over the different factors examined, while Mutatina et al. [27] scope the kinds of evidence available to health decision-makers in Uganda.

This evaluation of the 3DE programme faced limitations in terms of the ex-post design and its timing in relation to the interventions. However, the theory-based approach, combined with triangulation of different data sources derived from mixed methods, and a collaborative relationship between the evaluation specialists based in the programme implementing partner (CHAI), the commissioning group (UK Department for International Development) and the evaluation team resulted in rich results and engagement to absorb the lessons learned.

The evaluation concluded that 3DE had made a very limited contribution to changing evidence-based policymaking, capacity and behaviour in both countries. The main reasons behind this limited impact were judged to be two-fold. First, 3DE’s goal was inherently over-ambitious for a 3-year pilot. The overall goal, particularly in terms of building capacity and changing behaviour, requires a longer timeframe. Secondly, the programme had a number of aspirations that were not all compatible with one another. 3DE aimed to be demand-led, focused on robust impact evaluations, rapid, responsive and affordable, as well as catalysing action. A number of tensions or trade-offs exist within and between these aspirations. The overall lesson from the pilot is that even a very professional partnership cannot deliver on all of these in a short-term project in contexts like Zambia and Uganda, which are relatively typical of low- and middle-income settings. The necessary conditions for success are laid out in the revised theory of change (Additional file 1), which makes clear the many assumptions that need to be met for the desired outcomes to be reached. A key point arising from the political economy analysis was that the political economy of each issue selected for evaluation should influence how ‘progress’ is defined. For example, where the issue is strongly centrally driven, an evaluation may need to aim to affect policy decisions taken at a senior level. Where it is not, it may be more effective to focus on improving operational decisions and/or improving evaluative thinking and capacity more generally [25].

There therefore needs to be reflection on which objectives are most important and how to set realistic priorities. Different objectives – such as capacity building, brokering access to policy-relevant evidence, improving the supply of evidence, improving service delivery, and generating demand for evidence – imply different approaches and targets. The 3DE approach focused heavily on the characteristics of evaluations which supported or militated against evidence-based policy but less on the requirements of the evaluation users, whilst addressing the wider contextual challenges to evidence use. The evaluation findings reinforce the wider understanding that the demand for evidence varies substantially depending on individual policymaker attitudes, perceptions about the usefulness of evaluation evidence and credibility of the evaluator, awareness of evaluation benefits, technical skill in evaluation methods and the nature of the political system. Certainly, the presence of demand cannot be taken for granted and would need to be assessed on a case by case basis. It may sometimes be necessary to motivate demand for evaluation evidence through various strategies, such as the carrots, sticks and sermons (e.g. regulation, economic incentives and provision of information) [5].

It is also important to clarify what ‘demand-led’ really means. In the evaluation team’s view, the 3DE model was responsive to demand but until there is a much higher level of evaluative thinking and capacity within the MoH/MCDMCH, and a more strategic approach to establishing what evidence is needed in the short, medium and long terms, what 3DE provides is still effectively a supply-side activity. The demand for evidence “*encompasses both the capacity to find, evaluate and use…different forms of evidence and the motivation to use them to make evidence-informed policy*” ([25], p. 17), ensuring that decision-makers access and use a range of sources of evidence, not simply those they have directly commissioned. A priority for such programmes should be to increase evaluative thinking and capacity within local institutions to scope, oversee, quality assure and use evaluations – and more broadly to support what is now being termed ‘good governance of evidence’ [23]. Building capacity and motivation means strengthening individual skills, seeding new practices, learning by doing, sponsoring champions, building networks and supporting institutional processes [28]. There are some encouraging examples of government programmes aiming to do this – in South Africa, for example, the Department of Planning, Monitoring and Evaluation is building a demand-driven national evaluation system which ultimately aims to devolve responsibility for commissioning evaluations to departments [13, 17].

Whatever the focus chosen, the programme should be embedded in local institutions, with support provided externally as needed but with the key staff who are commissioning, providing, coordinating or brokering evidence being based within the Ministry or local research networks and organisations. This would also allow more flexibility about seizing policy ‘windows’, rather than having to identify them within the constraints of a short-term programme. Clearly, not all countries will have the same evidence needs and so a starting point for programming should be an understanding of the local institutional and market context, to understand what the gaps are and what existing institutions or networks could be strengthened, alongside the politics of the sector. In Zambia, for example, limited capacity and resources to use evidence in the Ministries, a neopatrimonial approach to policymaking generally, and a fragmented research supply market, heavily dependent on external funding, are some of the factors influencing how evidence is supplied and used [25].

The emphasis on ‘policy decisions’ or ‘policy changes’, which is common in externally funded programmes that want to demonstrate results, can also produce perverse effects. In the 3DE programme, contributing to a policy decision was a key performance target. The evaluation team found this problematic for a few reasons. First, this is a target that is hard to measure. There are so many influences on policymaking, many of which cannot be observed or disentangled, that isolating the contribution of evidence in shaping a decision is always problematic.

Secondly, while the emphasis on ‘policy decisions’ kept minds focused on the need to ensure take-up of research, there is also a potential conflict of interest between being a supplier of research and helping ministries to analyse and use evidence in a neutral way. Evidence may be presented to generate momentum for change, though that change may not be fully justified. The decision not to scale up a pilot programme (or to maintain the status quo in some way) also needs to be classified as a policy decision in this context, if this is what the evidence recommends.

In addition, if contribution to a policy decision is used as a target, then ‘policy decision’ should be broadened to include implementation, given the implementation gap which is so common in low-income countries [29]. A focus on more decentralised prioritisation, research and capacity-building at the district level would also be appropriate in many settings. Targets should also incorporate a focus on equity to avoid capture by more powerful interest groups, including better funded or organised programmes (such as the expense of less well-resourced areas, such as non-communicable diseases).

The evaluation also questioned the privileging of RCT-based impact evaluations as a higher form of knowledge. Different kinds of evidence are suited for different types of questions, and the 3DE modality could have focused on trying to generate or broker evidence that was suitable for the Ministry’s priority questions rather than privileging RCTs. RCTs are less suited to understanding processes, mechanisms, how and why implementation occurred, and the influence of context. Ministries rightly look for a range of information, including on equity and sustainability of interventions. Demand-generation or evidence-supply programmes should focus on supporting and providing appropriate tools for different questions. Evidence should fit with policy needs, so there should also be more flexibility about timeframes, for example. In some cases, having a longer time period would generate more useful and valuable information for the MoH than information generated rapidly. Overall value for money is also an important consideration. This was not assessed by the team and is itself a complex question [30]. However, the overall 3DE programme expenditure (including stages such as question sourcing and catalysation) was £400,000 per evaluation.

There is a broader question about the primary purpose of conducting evaluations. The 3DE approach viewed evaluations as tools for providing answers to policy questions. In this model, engagement with civil society and stakeholders is primarily a vehicle for improving the evidence to inform those answers. Yet, in Zambia, as in other low-income countries, civil society is generally weak. A complementary model of the evaluation process might view evaluations also as tools for engaging civil society and stakeholders around an issue, helping build local capacity to define and measure progress by creating robust and engaged evaluation processes. Thus, a stronger emphasis on the evaluation process, with the aim of building evaluative thinking and capacity, might help provide ‘answers’ that have a greater degree of legitimacy than evaluations that take a more extractive approach to citizen and stakeholder evidence.

## Conclusions

The evaluation of the 3DE programme in Uganda and Zimbabwe illustrates the complexity of the process of supporting evidence-based policymaking in healthcare, as in other sectors, and the need for clarity of focus and realism of expectations. The 3DE programme promised an ambitious set of targets, some of which were in tension with one another. It focused on producing evaluations whose characteristics would support evidence-based policymaking, while neglecting the wider context and the characteristics of evaluation users. A number of important lessons have been derived which are likely to apply in other low- and middle-income settings, such as the importance of supporting evaluative thinking and capacity within the wider institutions, of understanding the wider political economy of evidence use and its uptake, and of allowing for some flexibility in terms of programme targets. Fixating on one type of evidence is unhelpful in the context of institutions like ministries of health, which require a wide range of evidence to plan and deliver programmes. In addition, being tied to ‘policy decisions’ provides perverse incentives and neglects arguably more important aspects for evaluators, such as contributing to incremental programmatic adjustments and improved implementation.

## Additional file

Additional file 1: Extended theory of change. (PDF 471 kb)

## Abbreviations

3DE: demand-driven evaluations for decisions
CHAI: Clinton Health Access Initiative
CLEAR: Centres for Learning on Evaluation and Results initiative
MCDMCH: Ministry of Child Development Mother and Child Health
MoH: Ministry of Health
M&E: monitoring and evaluation
RCT: randomised controlled trial.
