# Supplementary material for: Generating demand for and use of evaluation evidence in government health ministries: lessons from a pilot programme in Uganda and Zambia
Source: Health Res Policy Syst. 2017 Oct 2;15:86. doi: 10.1186/s12961-017-0250-4 (PMC5625778; doi:10.1186/s12961-017-0250-4)
Supplement: Additional file 1: — Extended theory of change. (PDF 471 kb) [file 12961_2017_250_MOESM1_ESM.pdf]

Oxford Policy Management

In association with:

Independent evaluation of the Demand-Driven Impact Evaluation for Decisions (3DE) Pilot

Refined Theory of Change

July 2015

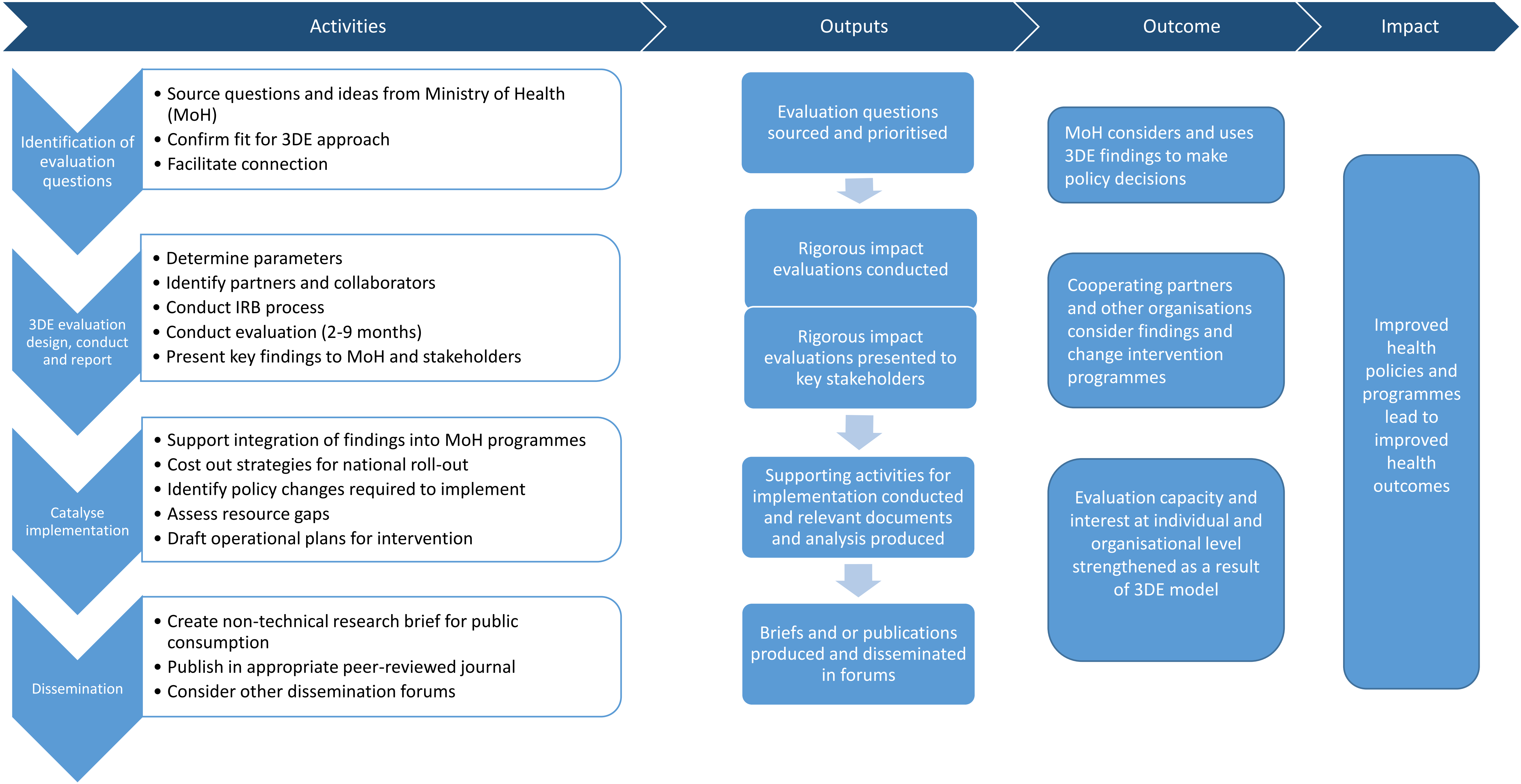
Activities
Outputs
Outcome
Impact
Identification of evaluation questions
• Source questions and ideas from Ministry of Health (MoH)
• Confirm fit for 3DE approach
• Facilitate connection
3DE evaluation design, conduct and report
• Determine parameters
• Identify partners and collaborators
• Conduct IRB process
• Conduct evaluation (2-9 months)
• Present key findings to MoH and stakeholders
Catalyse implementation
• Support integration of findings into MoH programmes
• Cost out strategies for national roll-out
• Identify policy changes required to implement
• Assess resource gaps
• Draft operational plans for intervention
Dissemination
• Create non-technical research brief for public consumption
• Publish in appropriate peer-reviewed journal
• Consider other dissemination forums
Evaluation questions sourced and prioritised
Rigorous impact evaluations conducted
Rigorous impact evaluations presented to key stakeholders
Supporting activities for implementation conducted and relevant documents and analysis produced
Briefs and or publications produced and disseminated in forums
MoH considers and uses 3DE findings to make policy decisions
Cooperating partners and other organisations consider findings and change intervention programmes
Evaluation capacity and interest at individual and organisational level strengthened as a result of 3DE model
Improved health policies and programmes lead to improved health outcomes

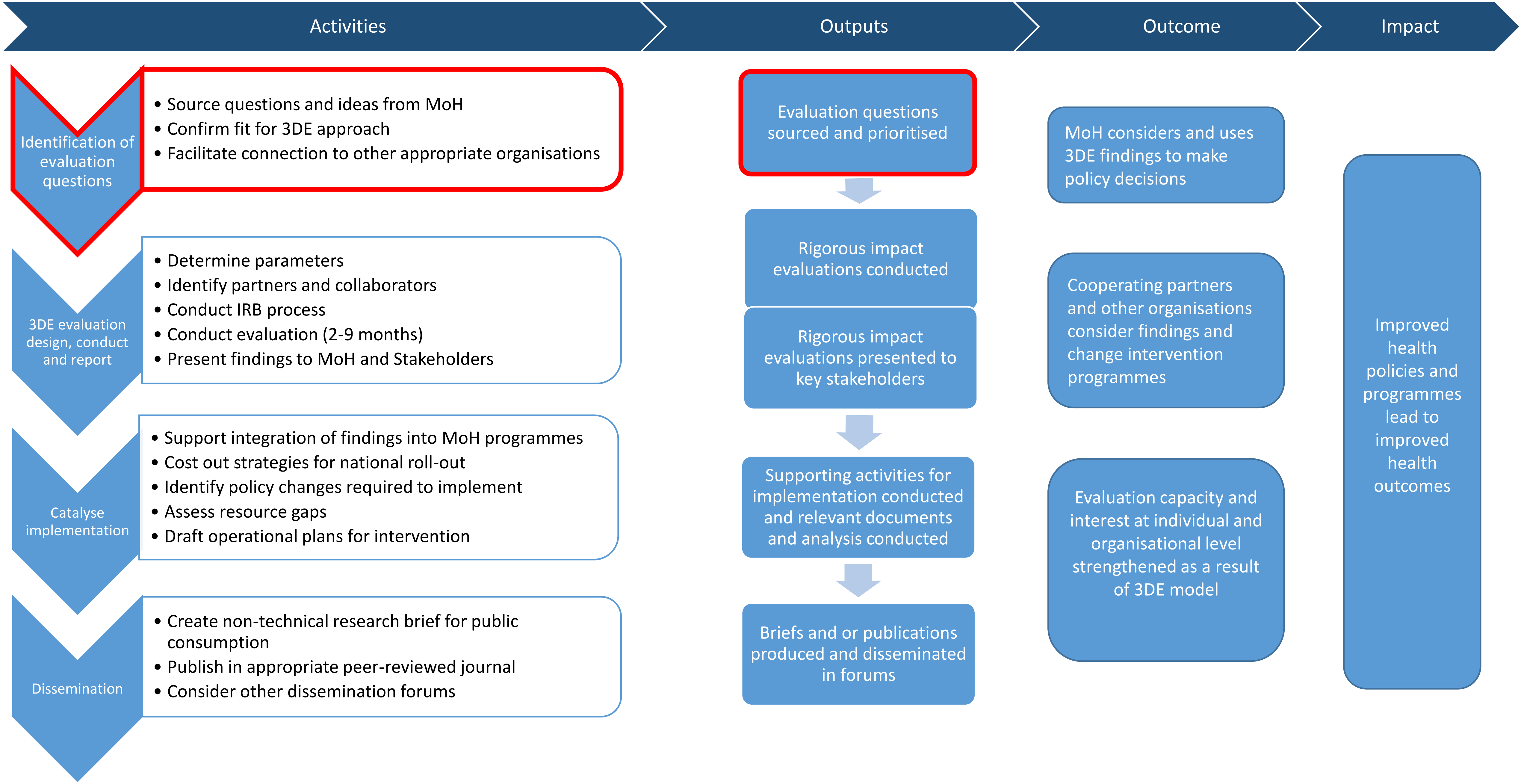
Activities
Outputs
Outcome
Impact
Identification of evaluation questions
• Source questions and ideas from MoH
• Confirm fit for 3DE approach
• Facilitate connection to other appropriate organisations
3DE evaluation design, conduct and report
• Determine parameters
• Identify partners and collaborators
• Conduct IRB process
• Conduct evaluation (2-9 months)
• Present findings to MoH and Stakeholders
Catalyse implementation
• Support integration of findings into MoH programmes
• Cost out strategies for national roll-out
• Identify policy changes required to implement
• Assess resource gaps
• Draft operational plans for intervention
Dissemination
• Create non-technical research brief for public consumption
• Publish in appropriate peer-reviewed journal
• Consider other dissemination forums
Evaluation questions sourced and prioritised
Rigorous impact evaluations conducted
Rigorous impact evaluations presented to key stakeholders
Supporting activities for implementation conducted and relevant documents and analysis conducted
Briefs and or publications produced and disseminated in forums
MoH considers and uses 3DE findings to make policy decisions
Cooperating partners and other organisations consider findings and change intervention programmes
Evaluation capacity and interest at individual and organisational level strengthened as a result of 3DE model
Improved health policies and programmes lead to improved health outcomes

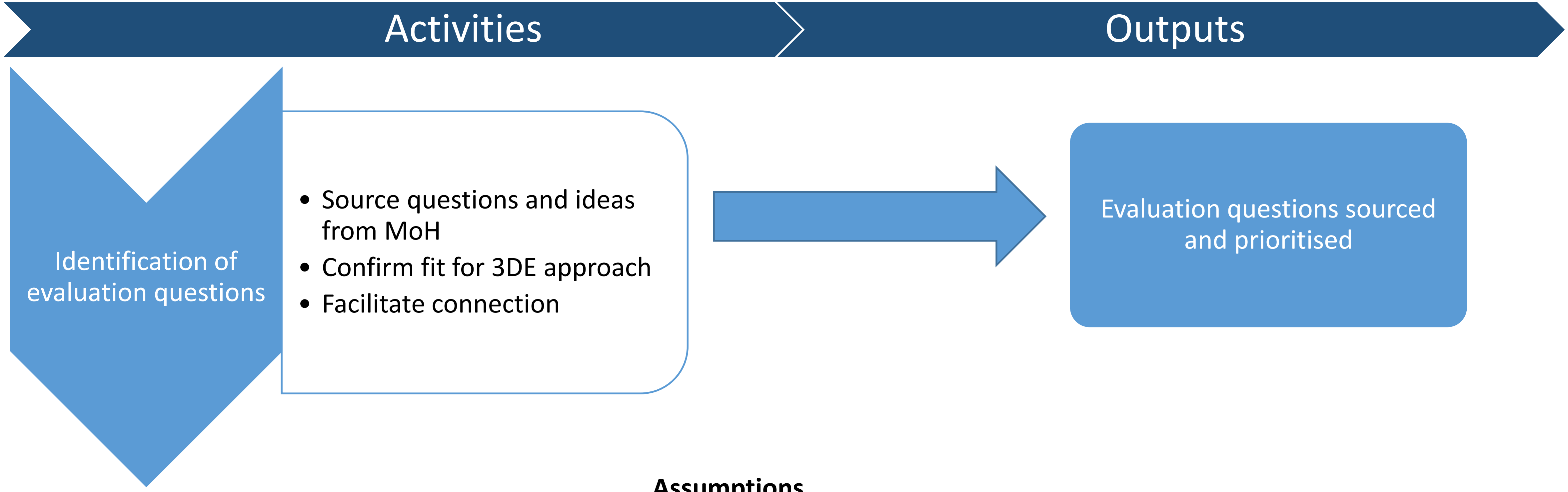

Contextual factor

- There is sufficient demand in MoH for evidence and in particular evaluations

Assumptions

- There is interest from Ministry to engage with sourcing of questions
- The Ministry has a number of questions it needs answers to (ideally situated in a wider research/evaluation policy)
- Good relations are established with appropriate sections within MoH
- Appropriate officials or units are identified and are part of question sourcing and prioritisation
- The Ministry is involved in developing, weighting and applying criteria for prioritisation
- Enough questions which meet the criteria exist
- The Ministry recognises chosen questions as relevant and high-priority

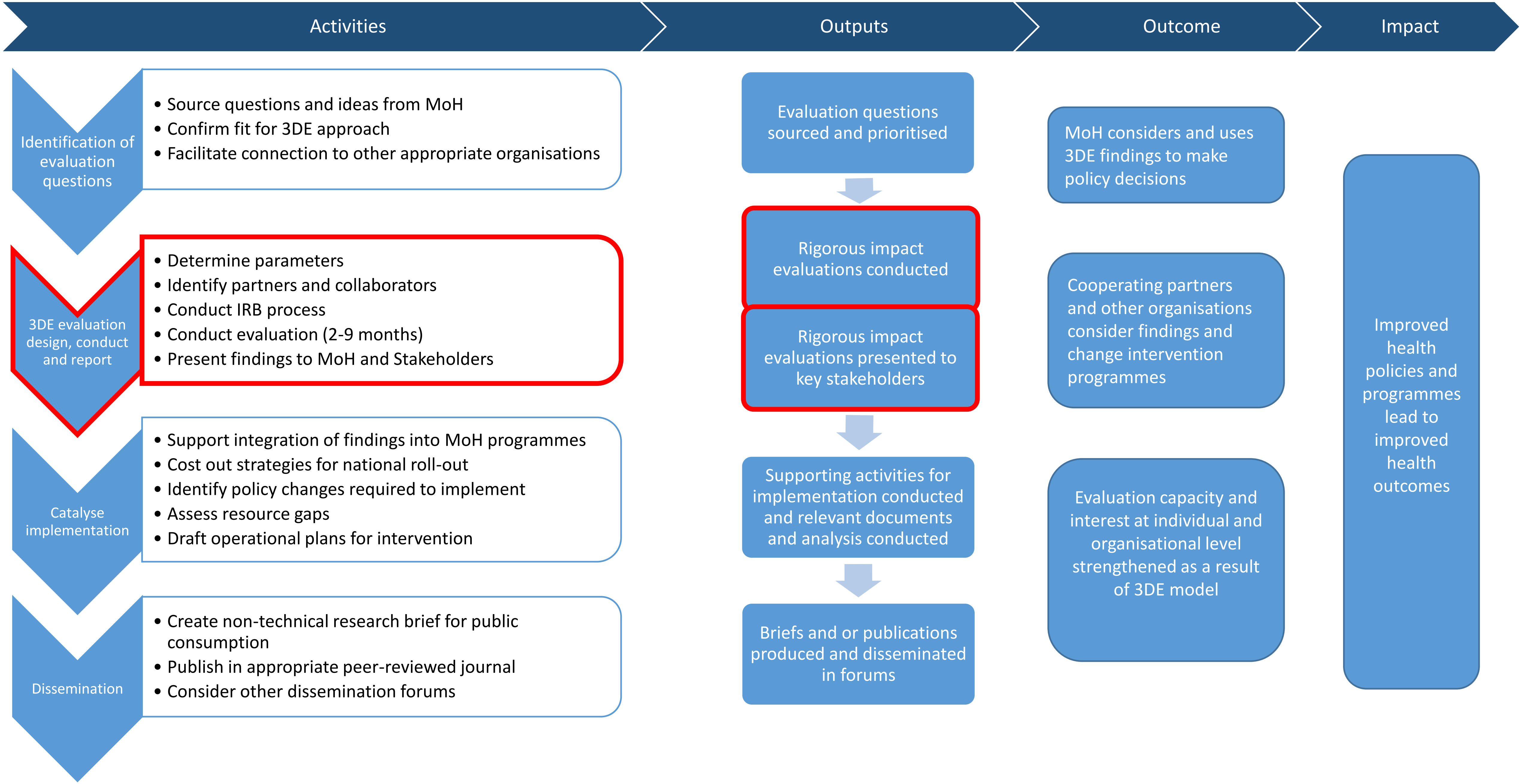
Activities
Outputs
Outcome
Impact
Identification of evaluation questions
• Source questions and ideas from MoH
• Confirm fit for 3DE approach
• Facilitate connection to other appropriate organisations
3DE evaluation design, conduct and report
• Determine parameters
• Identify partners and collaborators
• Conduct IRB process
• Conduct evaluation (2-9 months)
• Present findings to MoH and Stakeholders
Catalyse implementation
• Support integration of findings into MoH programmes
• Cost out strategies for national roll-out
• Identify policy changes required to implement
• Assess resource gaps
• Draft operational plans for intervention
Dissemination
• Create non-technical research brief for public consumption
• Publish in appropriate peer-reviewed journal
• Consider other dissemination forums
Evaluation questions sourced and prioritised
Rigorous impact evaluations conducted
Rigorous impact evaluations presented to key stakeholders
Supporting activities for implementation conducted and relevant documents and analysis conducted
Briefs and or publications produced and disseminated in forums
MoH considers and uses 3DE findings to make policy decisions
Cooperating partners and other organisations consider findings and change intervention programmes
Evaluation capacity and interest at individual and organisational level strengthened as a result of 3DE model
Improved health policies and programmes lead to improved health outcomes

Activities

Outputs

3DE evaluation design, conduct and report

- Determine parameters
- Identify partners and collaborators
- Conduct IRB process
- Conduct evaluation (2-9 months)

Rigorous impact evaluations conducted

Assumptions

- Evaluation designed, conducted and analysed according to established guidelines on quality and rigour
- Appropriate partners identified for implementation of the evaluation
- Funding and implementing partners are willing to implement programme in accordance with evaluation design and protocol
- Evaluation goes through ethical board and is approved in a timely manner
- The evaluation is conducted in a timely way to meet policy deadlines or windows of opportunity for scale-up, if applicable

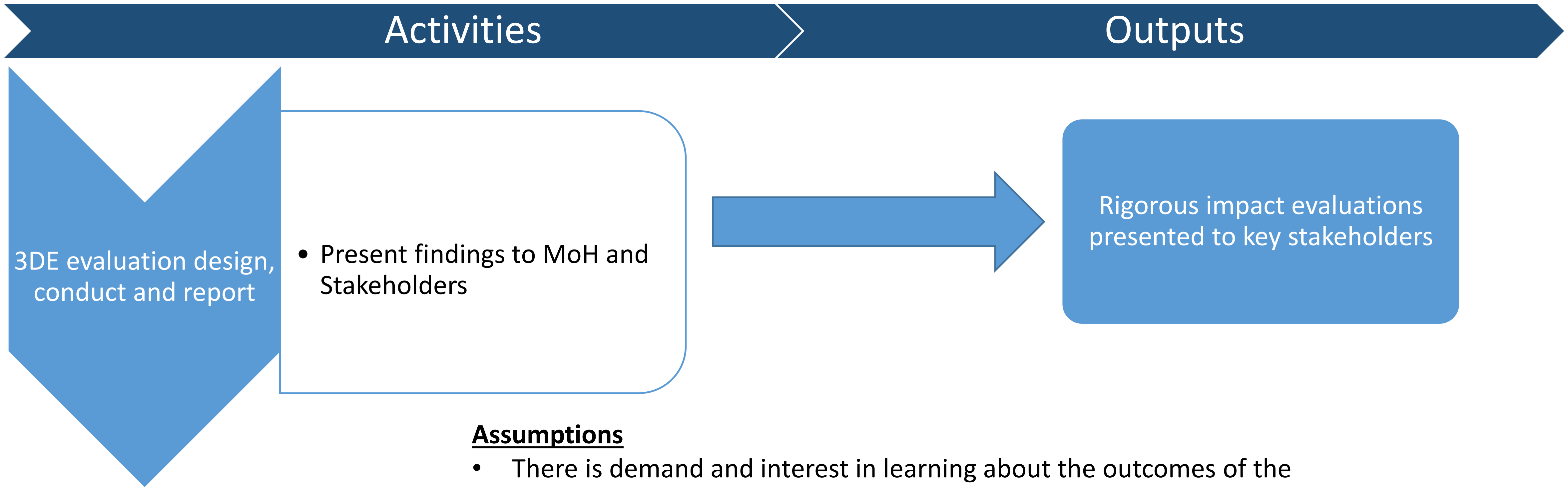

Assumptions

- There is demand and interest in learning about the outcomes of the evaluation by the main stakeholders
- Findings are presented to the right stakeholders
- The key stakeholders are present at the dissemination events organised and or fully informed and briefed
- The findings are presented in a accessible manner and tailored to the different audiences
- Presentations are robust, clear and accurately reflect data findings, strengths and limitations

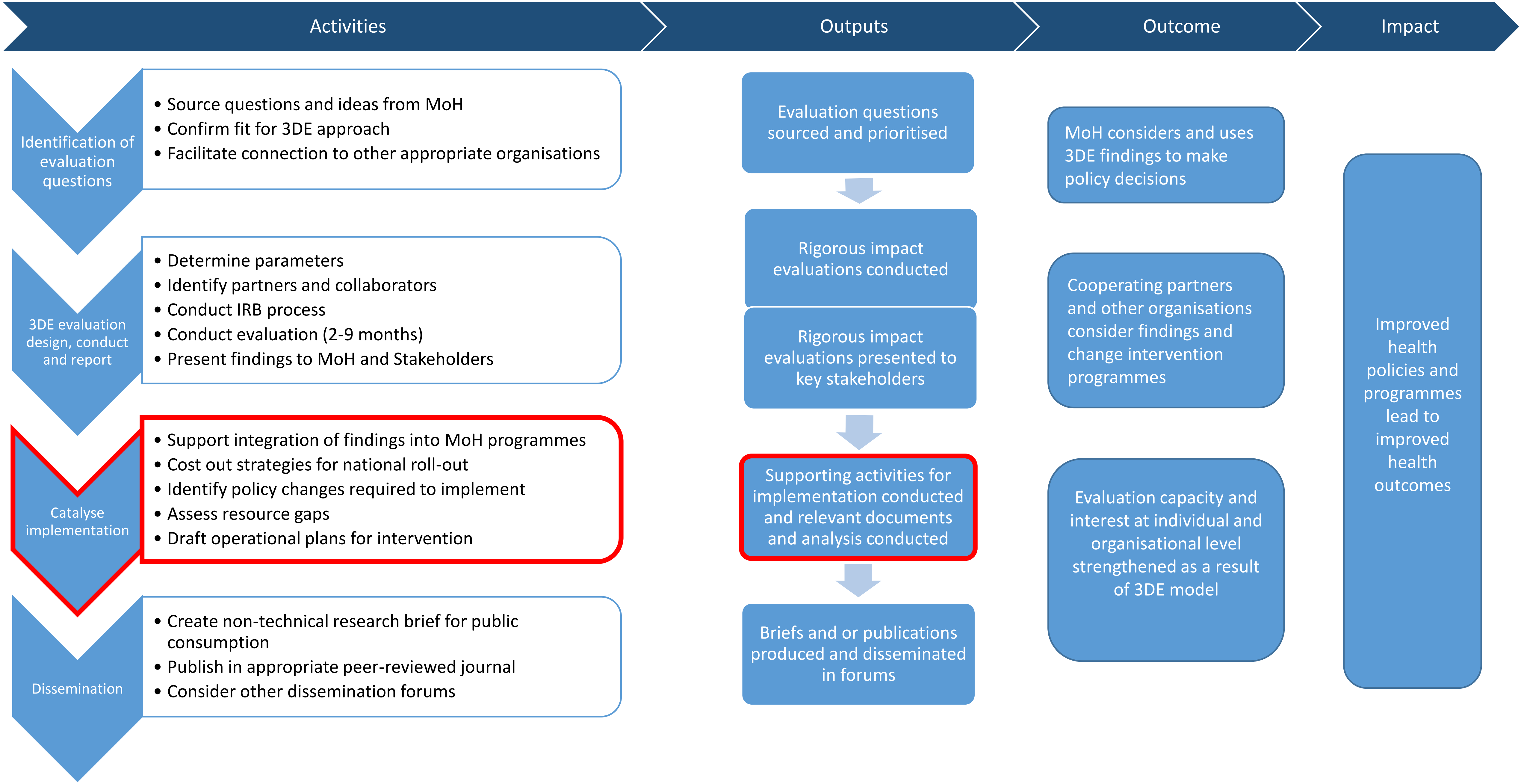
Activities
Outputs
Outcome
Impact
Identification of evaluation questions
• Source questions and ideas from MoH
• Confirm fit for 3DE approach
• Facilitate connection to other appropriate organisations
3DE evaluation design, conduct and report
• Determine parameters
• Identify partners and collaborators
• Conduct IRB process
• Conduct evaluation (2-9 months)
• Present findings to MoH and Stakeholders
Catalyse implementation
• Support integration of findings into MoH programmes
• Cost out strategies for national roll-out
• Identify policy changes required to implement
• Assess resource gaps
• Draft operational plans for intervention
Dissemination
• Create non-technical research brief for public consumption
• Publish in appropriate peer-reviewed journal
• Consider other dissemination forums
Evaluation questions sourced and prioritised
Rigorous impact evaluations conducted
Rigorous impact evaluations presented to key stakeholders
Supporting activities for implementation conducted and relevant documents and analysis conducted
Briefs and or publications produced and disseminated in forums
MoH considers and uses 3DE findings to make policy decisions
Cooperating partners and other organisations consider findings and change intervention programmes
Evaluation capacity and interest at individual and organisational level strengthened as a result of 3DE model
Improved health policies and programmes lead to improved health outcomes

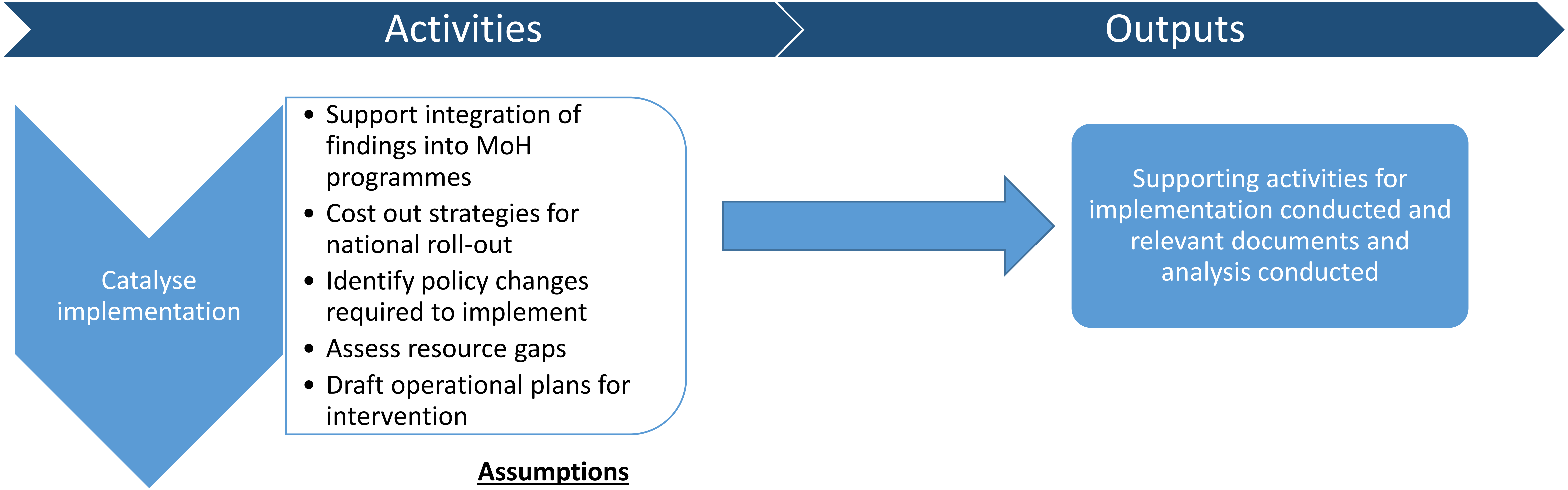

Assumptions

- The results have clear policy (or operational, if relevant) implications
- There are staff who are able to technically engage with the evaluations
- The ministry accepts the findings of the evaluation (takes ownership of findings)
- The ministry requires and requests for support (accepts proposed support)
- The support is provided on time and in accordance with the needs and requirements of the ministry
- The 3DE team is made up of individuals with the right technical skills and thematic areas of interest
- 3DE team engages with the right individuals and stakeholders within the ministry

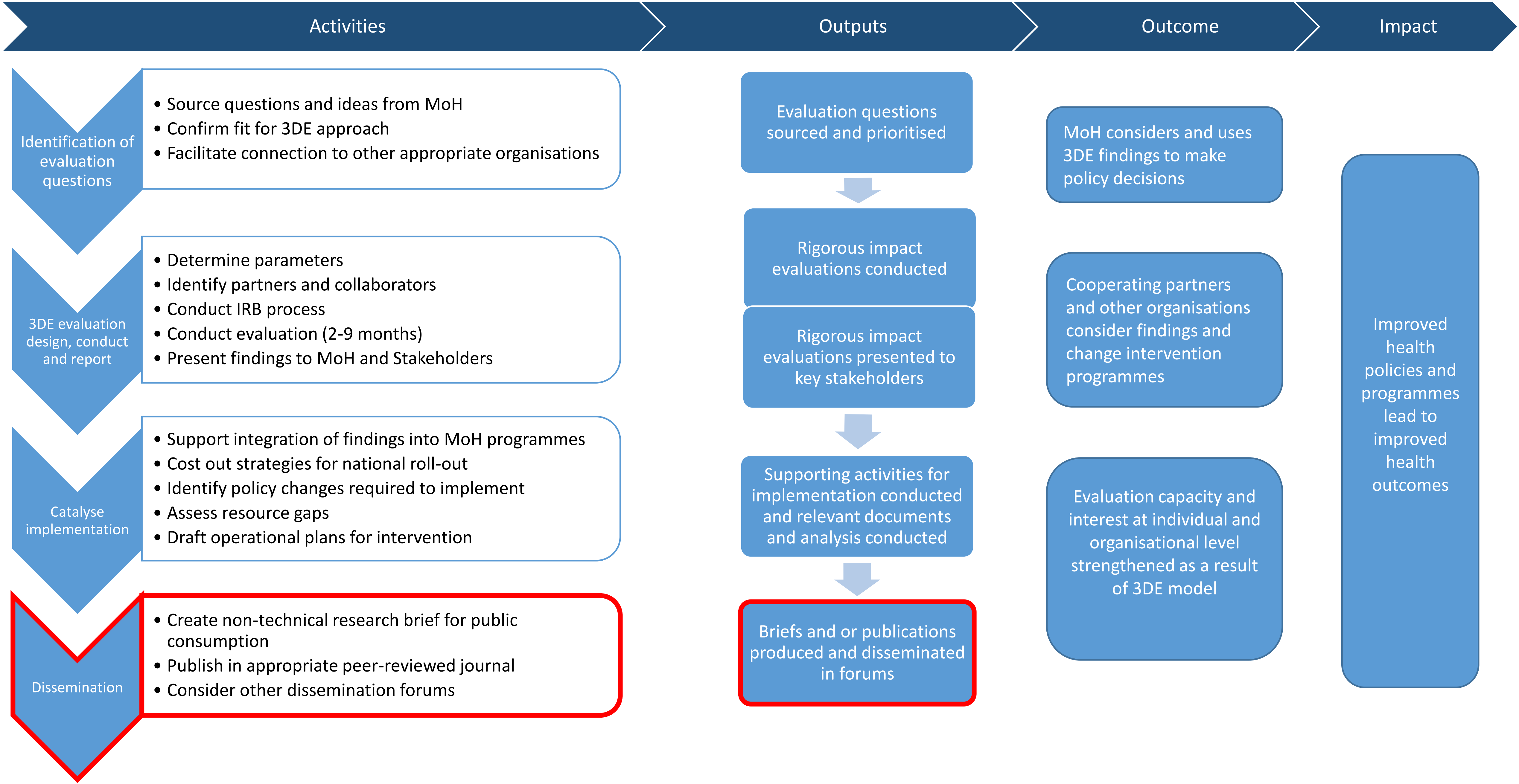
Activities
Outputs
Outcome
Impact
Identification of evaluation questions
• Source questions and ideas from MoH
• Confirm fit for 3DE approach
• Facilitate connection to other appropriate organisations
3DE evaluation design, conduct and report
• Determine parameters
• Identify partners and collaborators
• Conduct IRB process
• Conduct evaluation (2-9 months)
• Present findings to MoH and Stakeholders
Catalyse implementation
• Support integration of findings into MoH programmes
• Cost out strategies for national roll-out
• Identify policy changes required to implement
• Assess resource gaps
• Draft operational plans for intervention
Dissemination
• Create non-technical research brief for public consumption
• Publish in appropriate peer-reviewed journal
• Consider other dissemination forums
Evaluation questions sourced and prioritised
Rigorous impact evaluations conducted
Rigorous impact evaluations presented to key stakeholders
Supporting activities for implementation conducted and relevant documents and analysis conducted
Briefs and or publications produced and disseminated in forums
MoH considers and uses 3DE findings to make policy decisions
Cooperating partners and other organisations consider findings and change intervention programmes
Evaluation capacity and interest at individual and organisational level strengthened as a result of 3DE model
Improved health policies and programmes lead to improved health outcomes

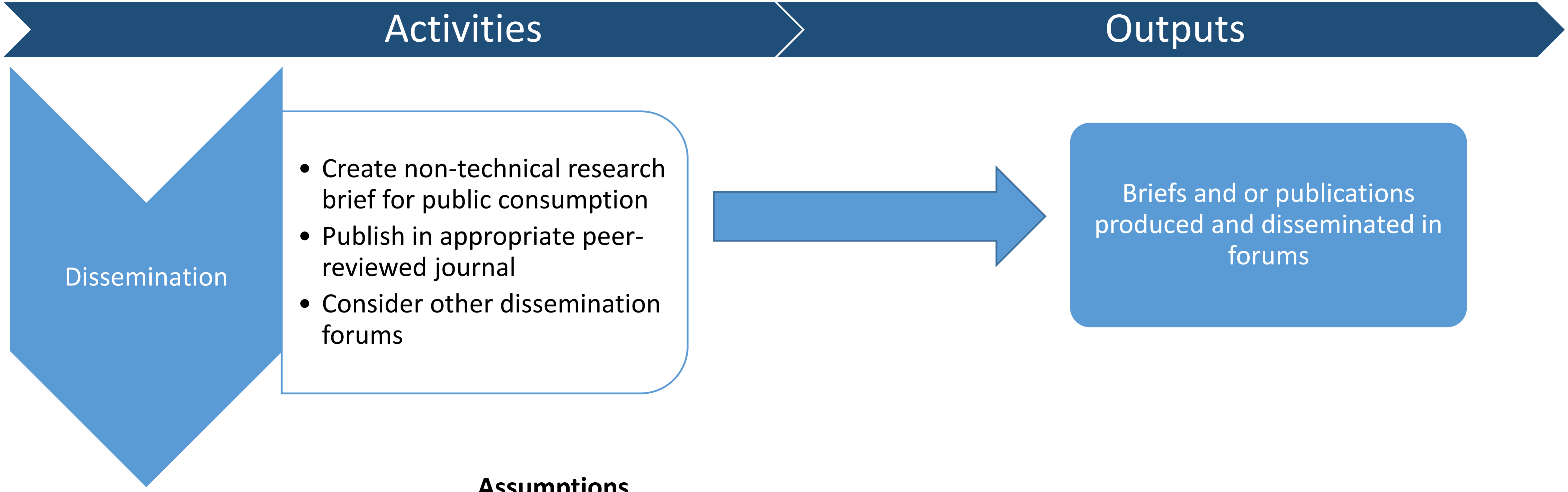

Assumptions

- The need for publication of non-technical research briefs identified
- Appropriate journal identified, papers produced and accepted by targeted journals
- Appropriate dissemination forums identified and 3DE team invited to attend

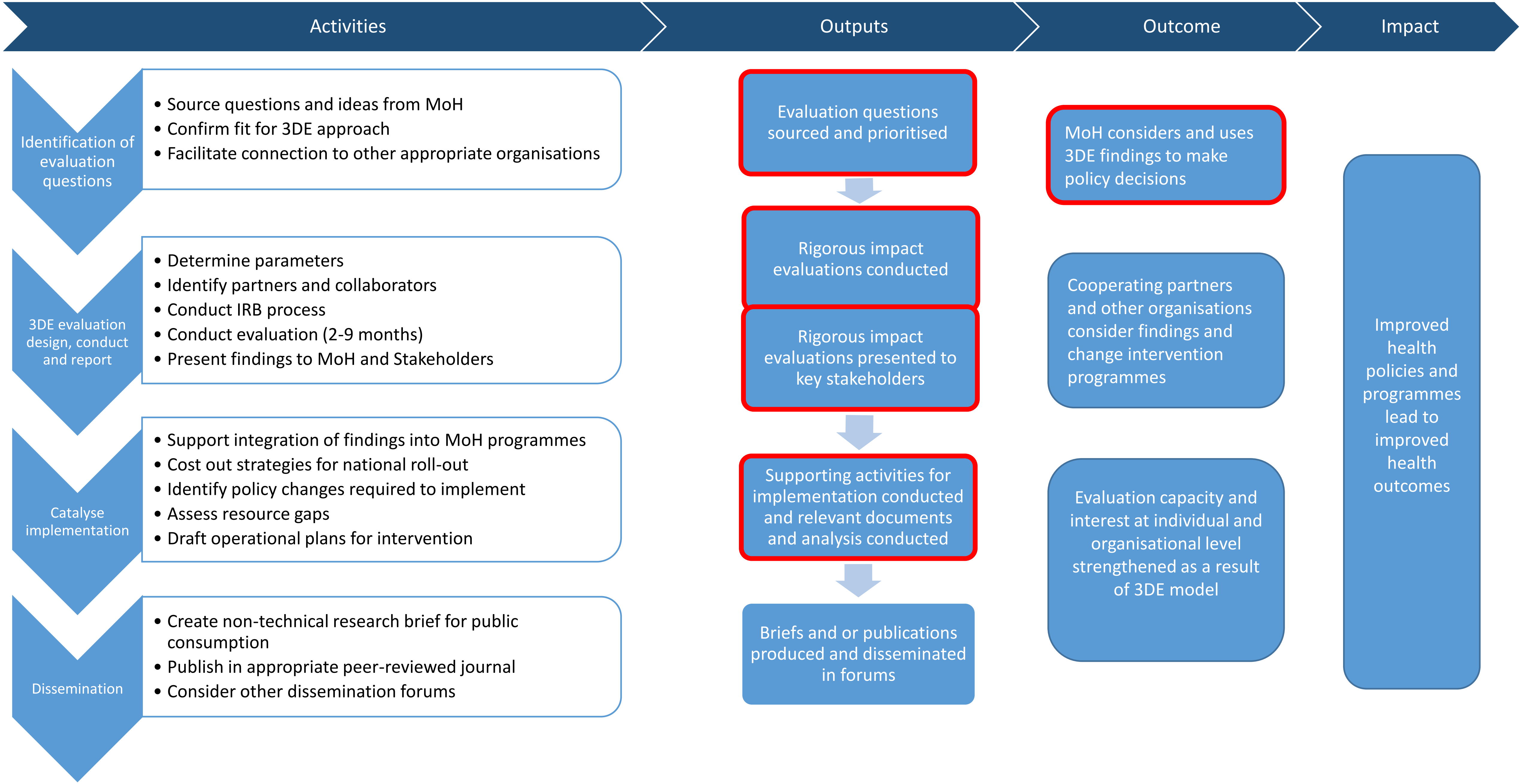

Activities
Outputs
Outcome
Impact
Identification of evaluation questions
• Source questions and ideas from MoH
• Confirm fit for 3DE approach
• Facilitate connection to other appropriate organisations
3DE evaluation design, conduct and report
• Determine parameters
• Identify partners and collaborators
• Conduct IRB process
• Conduct evaluation (2-9 months)
• Present findings to MoH and Stakeholders
Catalyse implementation
• Support integration of findings into MoH programmes
• Cost out strategies for national roll-out
• Identify policy changes required to implement
• Assess resource gaps
• Draft operational plans for intervention
Dissemination
• Create non-technical research brief for public consumption
• Publish in appropriate peer-reviewed journal
• Consider other dissemination forums
Evaluation questions sourced and prioritised
Rigorous impact evaluations conducted
Rigorous impact evaluations presented to key stakeholders
Supporting activities for implementation conducted and relevant documents and analysis conducted
Briefs and or publications produced and disseminated in forums
MoH considers and uses 3DE findings to make policy decisions
Cooperating partners and other organisations consider findings and change intervention programmes
Evaluation capacity and interest at individual and organisational level strengthened as a result of 3DE model
Improved health policies and programmes lead to improved health outcomes

Outputs

Rigorous impact evaluations conducted

Rigorous impact evaluations presented to key stakeholders

Supporting activities for implementation conducted and relevant documents and analysis conducted

Contextual factors

- Institutional and organisational structures provide incentives for demand for evidence and use
- The political environment allows for use of evidence in the policy process

Indicators of outcome being achieved

Expected to see

- Evaluation outputs presented in technical working groups (TWGs) policy development *(the work has something to say)*

Good to see

- TWG minutes show actions reference discussion of 3DE outputs
- Policy development process involves MoH ‘owners’ of 3DE evaluations

(the work is listened to throughout the policy development process)

Ideal to see

- Revised or new policy plans and budgets directly reference 3DE evaluation outputs (e.g. change can be attributed to 3DE contribution)

Outcome

MoH considers and uses 3DE findings to make policy decisions

Assumptions

- Sourced evaluation questions are still relevant to the ministry
- Evaluation findings are credible and accepted by the main stakeholders
- Evaluation findings are aligned with the political priorities and decisions of the ministry
- Evaluations conducted in timely manners and within the policy formulation timeframe of the ministry
- The policy formulation process is flexible to incorporate the findings of the evaluation
- Evaluations findings are clear, understandable and have clear policy implications
- The Ministry has resources to use for any changes to policy or programmes

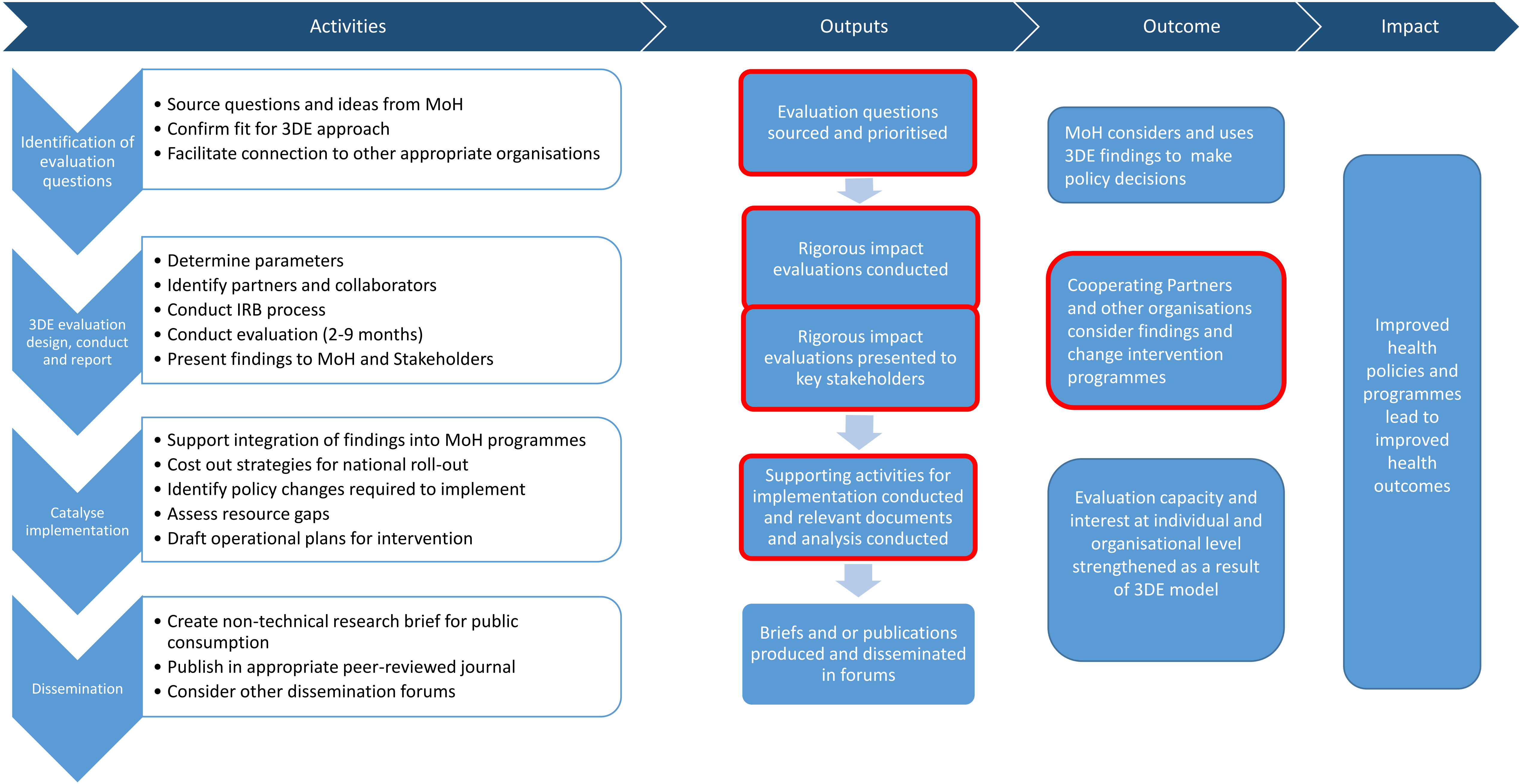

Activities
Outputs
Outcome
Impact
Identification of evaluation questions
• Source questions and ideas from MoH
• Confirm fit for 3DE approach
• Facilitate connection to other appropriate organisations
3DE evaluation design, conduct and report
• Determine parameters
• Identify partners and collaborators
• Conduct IRB process
• Conduct evaluation (2-9 months)
• Present findings to MoH and Stakeholders
Catalyse implementation
• Support integration of findings into MoH programmes
• Cost out strategies for national roll-out
• Identify policy changes required to implement
• Assess resource gaps
• Draft operational plans for intervention
Dissemination
• Create non-technical research brief for public consumption
• Publish in appropriate peer-reviewed journal
• Consider other dissemination forums
Evaluation questions sourced and prioritised
Rigorous impact evaluations conducted
Rigorous impact evaluations presented to key stakeholders
Supporting activities for implementation conducted and relevant documents and analysis conducted
Briefs and or publications produced and disseminated in forums
MoH considers and uses 3DE findings to make policy decisions
Cooperating Partners and other organisations consider findings and change intervention programmes
Evaluation capacity and interest at individual and organisational level strengthened as a result of 3DE model
Improved health policies and programmes lead to improved health outcomes

Outputs → Indicators of outcome being achieved → Outcome

Outputs

Rigorous impact evaluations conducted

Rigorous impact evaluations presented to key stakeholders

Supporting activities for implementation conducted and relevant documents and analysis conducted

Indicators of outcome being achieved

Expected to see

- Demand from co-operating partners for presentations of CHAI findings

(they value the work)

Good to see

- Co-operating partners bring CHAI findings to their meetings with MoH officials
- Co-operating partners reference CHAI findings in their own publications

(they use the work)

Ideal to see

- Co-operating partners report that CHAI findings have helped them influence policy development processes

(the work has made a difference)

Outcome

Cooperating partners and other organisations consider findings and change intervention programmes

Assumptions

- Development partners are interested in the evaluation questions
- Development partners are engaged with the 3DE process
- Evaluations and other outputs are disseminated to the Development partners
- The evaluation findings are credible for DPs
- The evaluation questions are relevant to the priority areas of Development partners or become priority areas for them
- The Development Partners have resources to consider changes in priorities or intervention programmes
- The Development Partners are flexible and able to respond to the findings of the evaluation

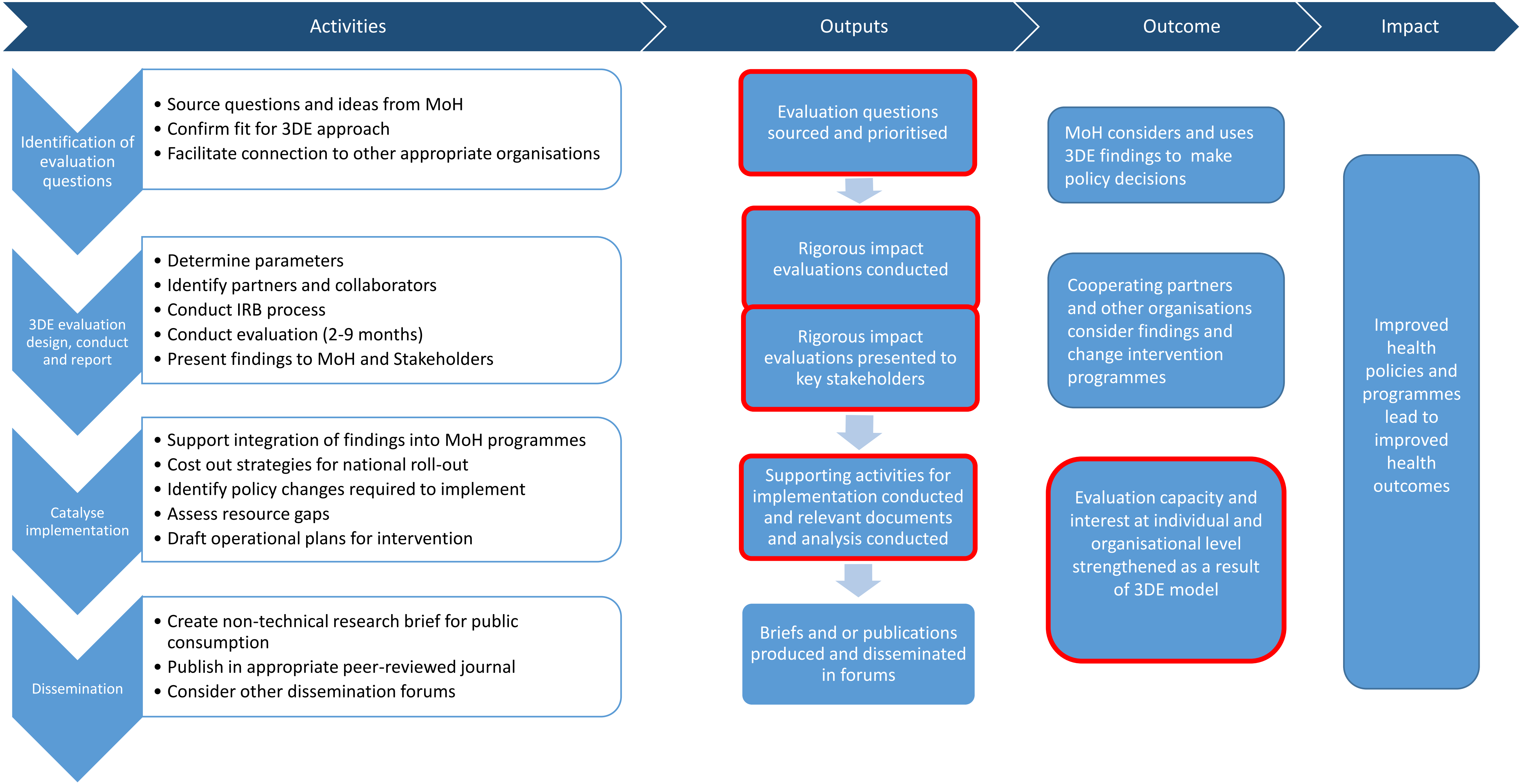
Activities
Outputs
Outcome
Impact
Identification of evaluation questions
• Source questions and ideas from MoH
• Confirm fit for 3DE approach
• Facilitate connection to other appropriate organisations
3DE evaluation design, conduct and report
• Determine parameters
• Identify partners and collaborators
• Conduct IRB process
• Conduct evaluation (2-9 months)
• Present findings to MoH and Stakeholders
Catalyse implementation
• Support integration of findings into MoH programmes
• Cost out strategies for national roll-out
• Identify policy changes required to implement
• Assess resource gaps
• Draft operational plans for intervention
Dissemination
• Create non-technical research brief for public consumption
• Publish in appropriate peer-reviewed journal
• Consider other dissemination forums
Evaluation questions sourced and prioritised
Rigorous impact evaluations conducted
Rigorous impact evaluations presented to key stakeholders
Supporting activities for implementation conducted and relevant documents and analysis conducted
Briefs and or publications produced and disseminated in forums
MoH considers and uses 3DE findings to make policy decisions
Cooperating partners and other organisations consider findings and change intervention programmes
Evaluation capacity and interest at individual and organisational level strengthened as a result of 3DE model
Improved health policies and programmes lead to improved health outcomes

Outputs

Rigorous impact evaluations conducted

Rigorous impact evaluations presented to key stakeholders

Supporting activities for implementation conducted and relevant documents and analysis conducted

Contextual factors

- Institutional and organisational structures provide incentives for demand for evidence and use
- The ministry has appropriate cadres and job profiles for individuals with strong research and evaluation skills and these positions are filled

Indicators of change in behaviour

Expected to see

- Interest from evaluation 'owners' in MoH to present evaluation findings at TWG meetings and meetings with co-operating partners *(3DE collaborators value the work)*

Good to see

- Demand for presentation of 3DE findings and methodology in other areas of MoH

(Others want to learn from the work)

Ideal to see

- Demand from other areas of MoH for 3DE evaluations

(Others demand more of the work)

- 3DE contributes to development / refinement of organisational strategy

(The demand is done in a strategic way)

Outcome

Evaluation capacity and interest at individual and organisational level strengthened as a result of 3DE model

Assumptions

- The 3DE model explicitly supports the development of individual and organisational skills and capabilities in commissioning evaluations, engaging in their implementation, and in understanding and interpretation of their findings through an elaborated plan of training/capacity building initiatives

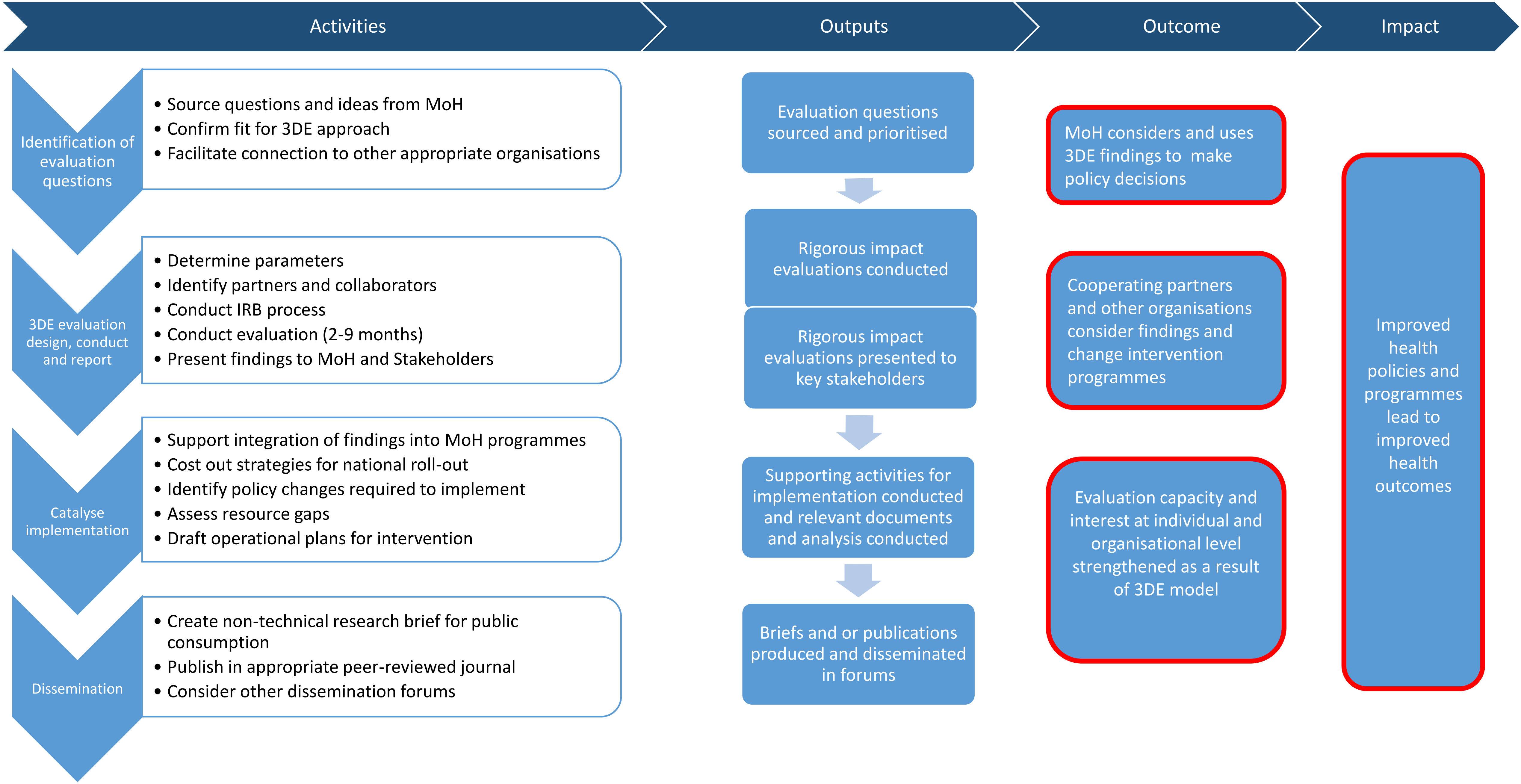
Activities
Outputs
Outcome
Impact
Identification of evaluation questions
• Source questions and ideas from MoH
• Confirm fit for 3DE approach
• Facilitate connection to other appropriate organisations
3DE evaluation design, conduct and report
• Determine parameters
• Identify partners and collaborators
• Conduct IRB process
• Conduct evaluation (2-9 months)
• Present findings to MoH and Stakeholders
Catalyse implementation
• Support integration of findings into MoH programmes
• Cost out strategies for national roll-out
• Identify policy changes required to implement
• Assess resource gaps
• Draft operational plans for intervention
Dissemination
• Create non-technical research brief for public consumption
• Publish in appropriate peer-reviewed journal
• Consider other dissemination forums
Evaluation questions sourced and prioritised
Rigorous impact evaluations conducted
Rigorous impact evaluations presented to key stakeholders
Supporting activities for implementation conducted and relevant documents and analysis conducted
Briefs and or publications produced and disseminated in forums
MoH considers and uses 3DE findings to make policy decisions
Cooperating partners and other organisations consider findings and change intervention programmes
Evaluation capacity and interest at individual and organisational level strengthened as a result of 3DE model
Improved health policies and programmes lead to improved health outcomes

Outcomes

Impact

MoH considers and uses 3DE findings to make policy decisions

Cooperating Partners and other organisations consider findings and change intervention programmes

Evaluation capacity and interest at individual and organisational level strengthened as a result of 3DE model

→

Improved health policies and programmes lead to improved health outcomes

Assumptions

- Policy decisions are implemented
- Financial resources are available for implementing policy decisions
- Health policies are improved (not just changed) as a result of the 3DE model
- Programmes are targeted at the appropriate population group and geographical area
- Programmes are implemented as planned
- Target populations respond to and utilise the programmes or interventions
- Predicted gains in terms of efficiency, equity and outcomes are realised (and any unintended negative consequences are limited)
- Where gains are made, such as increased efficiency, those are retained and reinvested in the health sector
